# Investigation of blood–brain barrier penetration and pharmacokinetics of a new formulation of cyanide antidote dimethyl trisulfide

**DOI:** 10.1007/s13530-025-00257-9

**Published:** 2025-04-29

**Authors:** Lóránd Kiss, Fruzsina R. Walter, Gábor Katona, Ana Raquel Santa-Maria, Ashley C. Whiteman, Christian T. Rios, Kyler D. Kelley, Breanna Nelson, David E. Thompson, Ildikó Csóka, Piroska Szabó-Révész, Mária A. Deli, Ilona Petrikovics

**Affiliations:** 1 Department of Chemistry, Sam Houston State University, 1003 Bowers Blvd, Huntsville, TX 77341 USA; 2 Department of Pathophysiology, University of Szeged, Szőkevalfi-Nagy Béla U. 6., Szeged, Hungary 6720; 3 Institute of Biophysics, Biological Research Centre, Szeged, Hungary; 4 Institute of Pharmaceutical Technology and Regulatory Affairs, University of Szeged, Szeged, Hungary; 5 Present Address: Wyss Institute for Biologically Inspired Engineering at Harvard University, Boston, MA USA

**Keywords:** Dimethyl trisulfide, DMTS, Cyanide antidote, Cyanide poisoning

## Abstract

**Objective:**

During cyanide poisoning, the rescue of vital organs like the brain is urgent. However, due to the presence of the blood–brain barrier (BBB), the currently available cyanide antidotes cannot reach the brain. Dimethyl trisulfide (DMTS) is a potent cyanide antidote and has excellent BBB permeability. Nonetheless, its formulation and application are challenging due to its highly lipophilic profile. In this work, a novel DMTS formulation, called FF-DMTS, was investigated. Its effect on in vitro DMTS permeability through BBB models, cellular viability, and in vivo absorption were tested.

**Methods:**

The particle size was measured in FF-DMTS formulation. The permeability of DMTS in this new formulation was tested in BBB-PAMPA and in primary triple co-culture models of BBB. The effect of FF-DMTS on cellular viability was determined. To test the membrane and barrier integrity transendothelial electrical resistance (TEER) and cell layer impedance measurements, immunofluorescent stainings and the fluorescein permeability technique were applied. The pharmacokinetics of DMTS were revealed in blood and brain tissue.

**Results:**

The average size of micelles in FF-DMTS was 16 nm. The permeability of DMTS through BBB-PAMPA and cell culture model was 7.68 × 10^–6^ and 23.81 × 10^–6^ cm/s, respectively. The FF-DMTS disturbed the barrier integrity of brain endothelial cells without causing any alteration in cellular viability until 300 µg/ml DMTS concentration. After administration of 150 mg/kg DMTS to mice, its absorption into the blood was rapid (5 min) and the plasma concentration of DMTS reached 5.2 µg/ml. The DMTS was also detected in brain, where its peak concentration was 495 ng/g brain tissue after 10 min of intramuscular administration. Furthermore, even 2 h later, DMTS was detected in brain.

**Conclusions:**

Here, we showed that the novel FF-DMTS formulation has good permeability through BBB and a remarkable pharmacokinetic profile. Therefore, further investigation of the efficacy of FF-DMTS for treating cyanide intoxication is important.

## Introduction

Cyanide (CN), the highly toxic chemical inhibits cytochrome c oxidase, the terminal oxidase of the mitochondrial electron transport chain, resulting in suppression of the cells’ oxygen utilization and aerobic ATP production, leading to lactic acidosis and eventually death [1, 2]. High energy-demand organs, like the brain and heart, are most vulnerable. Many industrial processes (gold mining, electroplating, and textile manufacturing) utilize CN, and it is also considered as a terrorist threat [3]. The presently utilized CN antidote Nithiodote ™ [4] has a sulfur donor component of sodium thiosulfate and a methemoglobin former scavenger component of sodium nitrite. The Cyanokit® [5] with its cobalt component similarly acts as a scavenger to antagonize CN. However, both have limitations, e.g., not enough efficacy, requirement of intravenous administration which prevents them from usage in mass casualty scenarios, or insufficient brain absorption because of the presence of blood–brain barrier (BBB) [6–8]. This drove the interest in developing newer antidotes, such as the sulfur donor type dimethyl trisulfide (DMTS) [3, 9] and the hydroxocobalamine-based Cyanokit® analog cobinamide [10]. DMTS has been approved and used for human as a flavor enhancer in food industry [11, 12]. DMTS was also investigated as a potential analgesic compound [13], and it has anti-inflammatory properties in acute pancreatitis (subcutaneous or intraperitoneal administration) or rheumatoid arthritis [14, 15].

Recent investigation efforts with DMTS are focused on developing various formulations for intramuscular administrations that are usable in scenarios of mass CN exposure [16]*.* Warnakula described the in vitro long-term stability [17]. As the heart and brain are among the most vulnerable organs during CN poisoning, pharmacological studies with DMTS should also focus on effective drug distribution in these tissues. Previous studies describe the in vitro & in vivo characterization of the polysorbate 80 formulated DMTS, its absorption kinetics and brain distribution along with the in vitro BBB penetration [18]. A new pharmacologically inert micellar-structured formulation of DMTS, labeled as FF-DMTS, with more promising absorption kinetics could allow a lower dose application to achieve the required antidotal effects. This recent study describes in vitro and in vivo characterizations for the newly formulated FF-DMTS as a CN antidote candidate. The DMTS permeability properties in BBB were determined, its effect on endothelial cell viability was assessed, and DMTS pharmacokinetics in blood and brain were measured after intramuscular administration.

## Materials and methods

### Chemicals

All chemicals were of the highest purity commercially available. DMTS, dimethyl disulfide (DMDS), sodium heparin from Sigma-Aldrich (St Louis, Missouri, USA), dibutyl disulfide (DBDS) from TCI America (Portland, Oregon, USA), HPLC grade distilled water from J.T. Baker (Center Valley, Pennsylvania, USA), Polysorbate 80 (Poly80; polyoxyethylene-sorbitan-20 mono-oleate or Tween 80) from Alfa Aesar (Ward Hill, Massachusetts, USA), HPLC grade acetonitrile, and ethanol from Acros Organics (Thermo Fisher Scientific, Waltham, Massachusetts, USA) were purchased. Heparin solution (10 U/ml) was prepared by diluting the 10 kU/ml heparin stock solution with 0.9% (w/v) saline solution. FF-DMTS was originated from the Southwest Research Institute (San Antonio, TX, USA), and the concentration of the stock solution was 100 mg/ml DMTS. DMDS was in acetonitrile at the concentration of 0.1 mg/ml, and DBDS was in ethanol at the concentration of 1 mg/ml. Ringer-HEPES buffer was prepared in distilled water resulting in the final concentrations of the components: 150 mM for NaCl, 2.2 mM for CaCl_2_, 0.2 mM for MgCl_2_, 5.2 mM for KCl, 5 mM for HEPES, 6 mM for NaHCO_3_, and 3.3 mM for glucose.

### Particle size distribution of FF-DMTS

The particle size influences the absorption of the active agents, and was determined for the FF-DMTS formulation. For viscosity limitations, dilution was necessary, therefore the examined samples contained 0.1 mg/ml DMTS. 1 ml of the sample in a polystyrene cuvette was loaded into a Zetasizer Nano (Malvern Panalytical, Worcestershire, UK) instrument which was used for the measurement. The DMTS refractive index was set to 1.602 and an absorption value of 0.001. The dispersant parameters for FF-DMTS formulation were the followings: temperature 25 °C, viscosity 0.620, and refractive index 1.367. For the measurement angle 173°, backscatter was selected.

### Testing FF-DMTS Permeability in the BBB-PAMPA System

A BBB—Parallel Artificial Membrane Permeability Assay (BBB-PAMPA) system was applied to model the passive permeability properties of the BBB and to test in vitro the diffusion of DMTS through the BBB [19]. The detailed method was published earlier [18]. Shortly, Prisma HT buffer was used as a solvent in the donor compartment and was diluted with HPLC purity water. The stock solution of 10 mg/ml FF-DMTS was diluted 100-fold with Prisma HT buffer to get the working solution of 0.1 mg/ml DMTS. Brain Sink Buffer (PN110674, pION) was used in the acceptor compartment. Before loading the solutions into the PAMPA plates, the concentrations of DMTS were determined by HPLC. Magnetic disks were added to the bottom (donor compartment) of a 96-well microplate (PN120551, pION). The wells of the plates were filled with 180 µl of 0.1 mg/ml DMTS. The BBB-PAMPA membranes on the acceptor plate (pION) were impregnated with 5 µl of BBB lipid cocktail (pION, PN 110672). The acceptor wells were filled with 200 µl Brain Sink Buffer. The plates were placed on the PAMPA plate stirrer (Gut-Box, pION), and the donor phase was stirred with 40 µm aqueous boundary layer. The DMTS samples from the acceptor phase were collected at 30 and 60 min, while the donor and acceptor phases were collected after a 90-min incubation (room temperature). HPLC–UV was used for analyzing the samples (detailed description is written later). The clearance volume was calculated for DMTS at 30, 60, and 90 min, and the apparent permeability was derived from the line fit to the clearance data (Kiss, Bocsik, et al. 2017; Kiss et al. 2014). For clearance calculation the following formula was applied:

$$Clearance\,\left(\mu L\right)= \frac{{[{C}_{t}]}_{Acceptor} \times {V}_{Acceptor}}{{[C]}_{Donor}}$$

The [C_t_]_Acceptor_ is the concentration of acceptor part at certain time point (30, 60, or 90 min); the [C]_Donor_ is the concentration of donor part at 0 min; the V_Acceptor_ is the volume of acceptor part.

### BBB cell culture model

The in vitro BBB model was built up from primary cultures of rat brain endothelial cells, glia, and pericytes as described previously [20, 21]. Endothelial cells and pericytes were isolated from 3-week-old Wistar rats. Mixed glial cultures (containing 90% astrocytes) were isolated from neonatal Wistar rats as described in detail by Nakagawa et al. [20]. In the case of cell culture experiments, all reagents were purchased from Merck Life Science Ltd. (Hungary), unless otherwise indicated. Brain endothelial cells were cultured in DMEM F-12 (Gibco, Life Technologies, Carlsbad, California) supplemented with 15% plasma-derived bovine serum, 100 µg/ml heparin, 5 µg/ml insulin, 5 µg/ml transferrin, 5 ng/ml sodium selenite, 1 ng/ml basic fibroblast growth factor (Roche, Switzerland), and 50 µg/ml gentamycin. The BBB model was established in the following manner: brain microvascular pericytes were passaged to the bottom side of 12-well tissue culture inserts (Transwell, polycarbonate membrane, 0.4 µm pore size, Corning Costar, USA) coated with collagen IV at a density of 1.5 × 10^4^ cells/cm^2^. After attachment of the pericytes, brain endothelial cells (8 × 10^4^ cells/cm2) were seeded to the upper side of the fibronectin and collagen IV coated membranes. Primary cultures of rat glial cells were passaged to the bottom of 12-well dishes (Corning, Costar, New York) coated with 100 µg/ml collagen type IV in sterile distilled water and cultured for 2 weeks before using for the triple co-culture model. Pericytes and glial cells were cultured in DMEM/ HAM’s F-12 supplemented with 10% fetal bovine serum (PanBiotech GmbH) and 50 µg/ml gentamycin. To construct the in vitro BBB co-culture model, Transwell culture inserts were placed into 12-well plates containing glial cells with endothelial culture medium in both compartments. After 2 days of co-culture leading to the formation of a confluent monolayer of brain endothelial cells, 550 nM hydrocortisone was added to the culture medium to tighten junctions. This triple co-culture model was used for transendothelial electrical resistance (TEER) measurement, permeability, and immunofluorescence experiments. Cellular viability experiments were performed on primary brain endothelial culture without pericytes and glial cells.

### Cell viability assays

Different viability assays were performed on brain endothelial cells with FF-DMTS treatment in Ringer-HEPES. Cellular metabolic activity (3-[4,5-dimethylthiazol-2-yl]-2,5-diphenyl tetrazolium bromide, MTT assay) and membrane integrity (lactate dehydrogenase, LDH assay) of brain endothelial cells were measured after 10 min FF-DMTS treatment in the 1–1000 µg/ml concentration range. For the detailed description of these assays see our earlier publications [22–24]. Briefly, based on the metabolic activity, the cells can convert the yellow MTT dye to purple formazan crystals, which can be determined by absorption measurements at 570 nm. In the case of the LDH assay cell culture, supernatant was collected at the end of the treatment. The extracellular LDH reflecting to membrane damage was determined by a kit according to the manufacturer’s instructions.

Using the real-time cell electronic sensing method (xCELLigence system, Roche), we could monitor cell viability, adherence, and barrier integrity continuously [25, 26]. This method utilizes impedance derived from an interaction between cells and electrodes of special 96-well plates. Background readings were performed with the culture medium, then endothelial cells were dispensed at the density of 5 × 10^3^ cells/well. Cells were cultured until reaching confluency, then treatments were started. Impedance was measured every 20 min. The cell index (CI) at each time point was defined as (R_n_ − R_b_)/15, where R_n_ is the cell-electrode impedance of the well when it contains cells, and R_b_ is the background impedance of the well with the medium alone. Normalization of the CIs was performed to the latest time point before the treatment of each group (CI_n_/ CI_before treatment_).

### Transendothelial electrical resistance measurement

The integrity of the paracellular barrier was determined by TEER measurement in triple co-culture cellular model of BBB. TEER measurements were performed with an EVOM Volt/Ohm Meter (World Precision Instruments, USA) combined with STX-2 electrodes and expressed relative to the surface area of the monolayers (Ω × cm^2^). The resistance of cell-free inserts (130 Ω × cm^2^) was subtracted from the measured values. Tight barrier integrity was measured on the brain endothelial cell layer before experiments (294 ± 30 Ω × cm^2^).

### Permeability measurements on triple co-culture models of BBB

The permeability of the FF-DMTS was also tested on the BBB triple co-culture model as it was described earlier [18]. Briefly, the cell culture was treated with 30 and 100 µg/ml FF-DMTS solution for 10 min. These concentrations are non-toxic to the cells. Transwell inserts were transferred to 12-well plates containing 1.5 ml Ringer-HEPES buffer in the acceptor (abluminal) compartments. In the apical side of endothelial cells, the culture medium was replaced with FF-DMTS treatment solution (donor compartment). After a 10-min incubation on a horizontal shaker, solutions from both compartments were collected and prepared for HPLC measurement. DMTS concentrations from the luminal and abluminal compartments were determined by HPLC–UV. After the penetration assay, the integrity of the brain endothelial barrier was verified by measuring the permeability of the marker molecule fluorescein (Mw: 376 Da; 10 µg/ml in R-H buffer). The assay with fluorescein was 15 min long. Concentrations of fluorescein in samples were determined by a fluorescence microplate reader (Fluostar Optima, BMG Labtechnologies, Germany; excitation wavelength: 485 nm, emission wavelength: 535 nm). The effective permeability coefficient (P_e_) for DMTS and fluorescein of DMTS were calculated as described in previous papers [27, 28].

### Immunohistochemistry

The morphology of brain endothelial cell junctions was investigated after immunostaining for zonula occludens-1 (ZO-1) protein. The Transwell inserts used in the permeability studies were washed with PBS, and the cells were fixed with 3% paraformaldehyde solution for 30 min at room temperature and incubated in 0.2% TX-100 solution for permeabilization for 15 min at 4 °C. 3% bovine serum albumin in phosphate buffered solution (PBS) was used to block the non-specific binding sites. Cells were incubated with primary antibodies rabbit anti-ZO-1 (Life Technologies, Carlsbad, California) overnight. After washing procedures, incubation with Alexa Fluor-488-labeled anti-rabbit secondary antibodies (Life Technologies, Invitrogen, USA) lasted for 1 h. The nuclei of the living cells were stained with Hoechst dye 33,342 for 5 min before fixation. Then, samples were mounted (Fluoromount-G; Southern Biotech, Birmingham) and staining was visualized by a Leica TCS SP5 confocal laser scanning microscope (Leica Microsystems GmbH, Wetzlar, Germany).

### Animals

CD-1 male mice were used for the animal studies (18–28 g; Charles River Breeding Laboratories, Inc., Wilmington, Massachusetts). Animal experiments were conducted in accordance with the guidelines of the Guide for the Care and Use of Laboratory Animals (National Research Council, 2010), accredited by AAALAC (American Association for the Assessment and Accreditation of Laboratory Animal Care, International). The mice were fed with 4% Rodent Chow (Teklad HSD, Inc., Madison, Wisconsin) and water ad libitum and were housed at 21 °C in light-controlled rooms (12-h light/dark, full-spectrum lighting cycle with no twilight). At the termination of the experiments, animals were euthanized in accordance with the AVMA Guidelines for the Euthanasia of Animals: 2013 Edition (AVMA Guidelines). The Institutional Animal Care and Use Committee (IACUC) permission number is 18-09-20-1015-3-01.

### Pharmacokinetics of FF-DMTS in blood and brain

For the absorption kinetics experiments, the DMTS concentration in blood and brain was measured from samples taken 0, 5, 10, 15, 30, 60, and 120 min after intramuscular injection. A 150 mg/kg DMTS dose was applied by injecting the 100 mg/ml FF-DMTS stock solution intramuscularly (right leg). For the control mouse, the vehicle was applied without DMTS. At the end of treatment, mice were deeply anesthetized by inhalation of isoflurane before the blood and the brain samples were taken. Then, blood samples were collected from the heart into heparinized tubes. After that blood was washed off by performing cardiac perfusion with approximately 8 ml physiological saline containing 10 U/ml heparin under the deep terminal anesthesia. Following a decapitation, the brain was quickly removed from the skull, and divided into two halves, from which one half was used in DMTS analysis, while the other half was frozen. The measurement of the DMTS concentrations in the blood and brain samples was initiated immediately following collection to minimize the effect of any post-collection reactions of DMTS with the sample [29].

### DMTS samples preparation for HPLC analysis

*DMTS samples from the BBB-PAMPA experiments*. The DMTS concentrations from the R-H solutions in the BBB-PAMPA system were determined by HPLC–UV. A 60-µL aliquot of internal standard solution (0.05 mg/ml DMDS in acetonitrile) was transferred to 250 µl glass inserts within the HPLC glass vials (Agilent Technologies), and 40 µl of the BBB-PAMPA DMTS samples were added to each insert. The vials were hand-vortexed for 10 s, followed by auto-vortexing for 5 min at room temperature, and loaded into the HPLC instrument for measurement.

*DMTS samples collected from the cell culture measurements*. A 300-µl aliquot of internal standard solution (0.05 mg/ml DMDS in acetonitrile) was mixed with 200 µl of the DMTS containing samples. The solutions were hand-vortexed for 10 s, auto-vortexed for 5 min at room temperature, and centrifuged for 5 min at 4 °C with 14 000 RCF. The supernatant (150 µl) was transferred to 250 µl glass inserts in the HPLC glass vials, and loaded into HPLC instrument for measurement.

*Whole blood samples collected from animals*. The detailed blood preparation method was described earlier [29]. Briefly, immediately after collection of the blood (80 µl) from DMTS-treated animals, 200 µl of the internal standard (0.1 mg/ml DMDS) in ice cold acetonitrile was added. Then, the microcentrifuge tubes with the solutions were hand-vortexed for 10 s, auto-vortexed for 10 min at room temperature, and centrifuged for 5 min at 4 °C and 14 000 RCF. The supernatants (80 µl) were transferred to 250 ml glass inserts in the HPLC glass vials, and loaded into HPLC instrument for measurement.

### DMTS analysis by HPLC–UV

A Dionex Ultimate 3000 (Thermo Scientific, Waltham, Massachusetts) HPLC–UV instrument was used in the analysis [29]. Forty microliters of DMTS containing samples were injected into a guard column connected to a 250 × 4.60 mm nonpolar C-8 analytical column having a Phenomenex Luna stationary phase (consisting of bonded octane units coated on silica support particles, pore size of 100 Å, outer diameter of 5 mm). Isocratic elution was employed with a 35:65 v/v mixture of water and acetonitrile flowing at the rate of 1 ml/min served as the mobile phase. The column backpressures ranged from 1430 to 1450 psi. The analyte absorbance at 215 nm was monitored by a UV detector. This HPLC–UV method was also used for experiments with blood.

### DMTS analysis by gas chromatography–mass spectrometry

Gas chromatography–mass spectrometry (GC–MS) was used to measure DMTS concentration from the brain samples with a previously developed method [29]. To every 220-mg mouse brain tissue, 1 ml ethanol was added. The brains were homogenized by a Precellys 24 tissue homogenizer (Bertin Technologies, Montigny-le-Bretonneux (France), Precellys vials with 1.4 mm ceramic beads, 6500 RPM, 3 times 1 min). The brain homogenate (475 ml) was spiked with 25 µl of 1 mg/ml DBDS (in ethanol) solution. A magnet bar was placed into the vials and the sample was stirred for 5 min. SPME-PDMS fiber (Agilent Technologies) was inserted into the headspace of the vials and was incubated for 10 min.

For sample analysis, an Agilent Model 6890 A gas chromatograph and Agilent Model 5973 C mass selective detector were used. The GC column was an Agilent DB-5MS (30 m × 0.25 mm with 0.1 mm film). The chromatographic method parameters were: 40 °C for 1 min, 60 °C/min to 280 °C, and 280 °C for 3 min with a He flow rate of 1 ml/min. The temperature of the inlet was 250 °C. The source temperature for MS was 230 °C, while the quadrupole temperature was 150 °C. Both scan mode and single ion monitoring (SIM) were applied for detection, in the scan range between 30 and 200 m/z. The following ions were selected for the SIM detection with 5 ms dwell time for DMTS: 44.9, 45.0, 63.9, 64.0, 78.9, 79.0, 110.8, 111.0, 125.9, and 126.0 m/z. The ions used for DBDS quantification included: 178 and 178.1 m/z. For data processing, Agilent ChemStation version E.02.02.1431 software and OpenChrom (Lablicate GmbH, Hamburg, Germany) were used. Concentrations were determined by the calibration curve published earlier, and the LOD and LOQ were determined to be 213 and 645 ng DMTS/g brain, respectively (Kiss, Holmes, et al. 2017). The intra- and inter-day precisions were below 24.3 CV %, while the accuracy was between −1.3% and + 2.4%.

### Statistical analysis

All plotted values represent the means ± standard error (SEM). GraphPad Prism 5.0 (GraphPad Software Inc., San Diego, California) software was used for statistical analysis. Unpaired Student t-test was used to assess the significance of changes in the permeabilities of sodium fluorescein or DMTS. To evaluate the toxic effect of DMTS on brain endothelial cells, one-way ANOVA followed by Dunnett’s test was used. Two-way ANOVA followed by Dunnett’s test was performed in case of TEER measurements. The effect of the variable under study was considered statistically significant, if the random probability (p) of the observed change in signal associated with a specific treatment was < 0.05. The number of replicate samples varied from 3 to 10.

## Results

### Particle size distribution for FF-DMTS and its permeability through BBB-PAMPA system

**Fig. 1:**
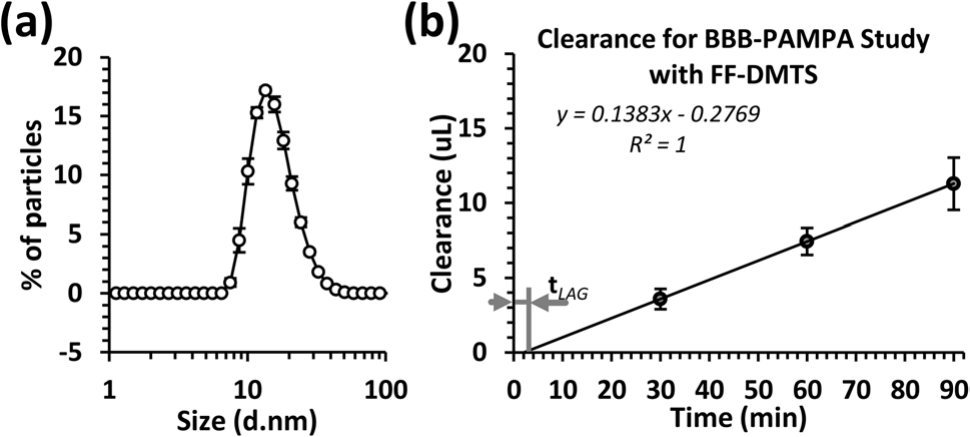
**a** Particle size distributions in FF-DMTS. **b** Clearance volume for FF-DMTS in the BBB-PAMPA system. The clearance volume of DMTS was plotted against time. Data are presented as mean ± SEM (*n* = 3)

The particle size distribution of FF-DMTS micellar formulation was measured (Fig. 1(a)). The FF-DMTS resulted in an average particle size of 16.0 nm, and the range of particle size was between 7.5 and 38 nm.

The BBB-PAMPA is a well-characterized surrogate model for BBB [19, 30, 31]. By using this tool, the permeability of FF-DMTS was estimated through BBB. Based on the measured DMTS concentration values at 30, 60, and 90 min after the start point, the clearance was calculated and presented in Fig. 1(b). The lag time (t_*LAG*_), which is necessary for the DMTS to cross the BBB-PAMPA membrane, was 2.002 min. The P_app_ of FF-DMTS in PAMPA system was 7.68 × 10^–6^ cm/s.

### The effect of FF-DMTS on endothelial cell viability

**Fig. 2:**
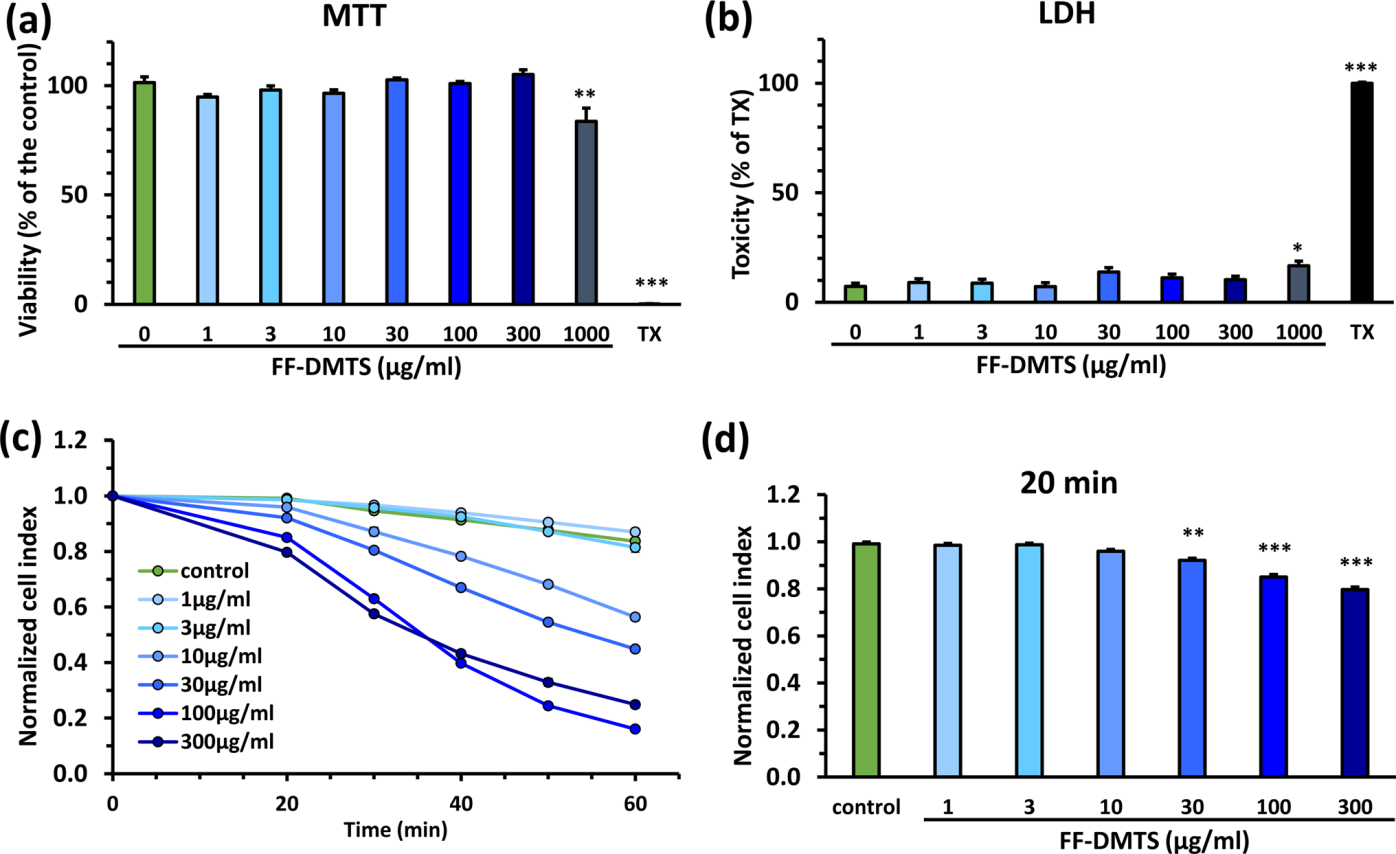
FF-DMTS viability experiments on primary rat brain endothelial cells. The effect of FF-DMTS on cellular viability after 10 min treatment in Ringer-HEPES buffer was measured by **a** MTT test or **b** LDH assay. **c** and **d** Real-time cell electronic sensing measurement. Cell index is calculated from the impedance of cell layers which reflects adherence, barrier integrity, and viability of the cultured brain endothelial cells. Due to the treatment duration on the 96-well plate, the first measured time point was 20 min. **c** Kinetics of impedance until 60 min; **d** Normalized cell index values at 20-min time-point. Abbreviations: TX = Triton X-100 detergent as 100% cell death control. MTT: 3-[4,5-dimethylthiazol-2-yl]-2,5-diphenyl tetrazolium bromide; LDH: lactate dehydrogenase. One-way ANOVA with Dunnett’s post-test;*, *p* < 0.05; **, *p* < 0.01; ***, *p* < 0.001, compared to the control, *n* = 4–8

We performed viability assays to test the metabolic activity (MTT assay) and membrane integrity (LDH assay) of brain endothelial cells after 10 min FF-DMTS treatment in Ringer-HEPES solution in the 1–1000 µg/ml concentration range (Fig. 2). The 10 min duration was selected because, under in vivo condition, high concentration of DMTS can be detected for approximately this time. After that short (10 min) FF-DMTS treatment, we only saw metabolic activity change (MTT, Fig. 2a) or membrane integrity compromising (LDH, Fig. 2b) effects after treatment with the highest (1000 µg/ml) concentration. No other concentrations seemed to alter cell viability in these settings.

Using the real-time cell electronic sensing method, we monitored cellular impedance in real-time (Fig. 2c and d). In this measurement, the treatments were not stopped after 10 min but were followed through 1 h (Fig. 2c). The results were expressed as normalized cell index. We observed that the 20 min treatment with 30 to 300 µg/ml concentrations caused a drop in the impedance (Fig. 2d). This effect was observed for lower concentrations (3–10 µg/ml) of DMTS at one-hour time-point without a recovery. This indicates a damaging effect on the brain endothelial cell viability, adherence, and barrier integrity which can be caused by DMTS and/or the vehicle.

### The effect of FF-DMTS on the barrier integrity of the primary rat triple co-culture model of BBB

**Fig. 3:**
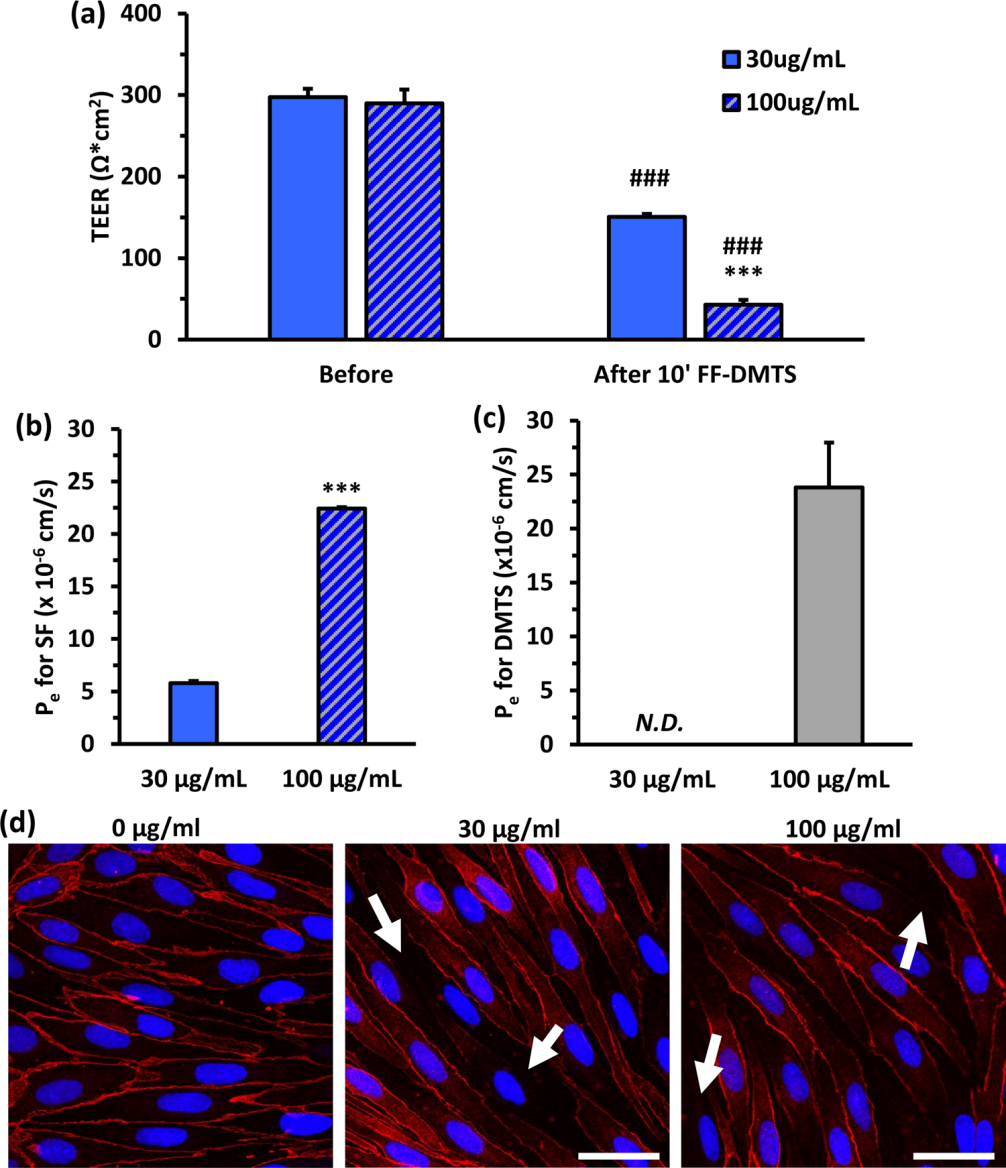
Barrier integrity and permeability measurements on the blood–brain barrier co-culture model after 10 min FF-DMTS treatments. **a** Transendothelial electrical resistance (TEER) was measured before and after the experiment. Two-way ANOVA with Dunnett’s post-test, ###, *p* < 0.001, compared to before treatment value; ***, *p* < 0.001, compared to the 30 µg/ml treatment concentration, *n* = 4. **b** Sodium fluorescein (SF) permeability was assessed after FF-DMTS treatment of the BBB model. P_e_: endothelial permeability coefficient. Unpaired t-test, ***, *p* < 0.001, compared to the control, 30 µg/ml treatment concentration, *n* = 4. **c** Permeability of DMTS through BBB co-culture model after treatments with 30 and 100 µg/ml FF-DMTS. N.D.: not detected. **d** Immunofluorescent images of zonula occludens-1 (ZO-1) junctional associated protein after FF-DMTS treatments (0, 30, 100 µg/ml) on primary brain endothelial cells. Bar: 100 µm

The effect of FF-DMTS on barrier integrity and DMTS permeability was investigated in the BBB cell culture model. The TEER was uniform and high in the BBB model at the beginning of the experiment (294 ± 30 Ω × cm^2^, Fig. 3a). After the 10 min FF-DMTS treatment, we observed a sharp TEER decrease. The 30 µg/ml FF-DMTS treatment resulted in a resistance of 151 ± 8 Ω × cm^2^, which represents 50% of the original untreated value. In the case of the 100 µg/ml concentration, this decrease was even larger: the TEER dropped to 43 ± 14 Ω × cm^2^, which represents 14% of the original untreated value (Fig. 3a). We confirmed these findings with the measurement of fluorescein permeability (Fig. 3b). The endothelial permeability coefficient (P_e_) was in the normal range of 5.8 ± 0.4 × 10^–6^ cm/s after treatment with 30 µg/ml FF-DMTS for 10 min. However, the higher treatment concentration (100 µg/ml) resulted in a four-time elevation of P_e_ (22.4 ± 0.3 × 10^–6^ cm/s) for fluorescein, reflecting a leaky barrier. The DMTS permeability was also measured after treatment with FF-DMTS at 30 and 100 µg/ml concentrations (Fig. 3c). Unfortunately, the DMTS molecule could not be detected after 30 µg/ml FF-DMTS treatment in the acceptor compartment. However, the P_e_ for 100 µg/ml FF-DMTS treatment was 23.81 ± 4.16 × 10^–6^ cm/s.

The immunohistochemistry for the linker protein ZO-1, associated with tight interendothelial junctions, was also performed (Fig. 3d). We found that in both cases (30 and 100 µg/ml of FF-DMTS for 10 min), the junctional staining became uneven between the cells. This is in accordance with the decrease in barrier integrity measured by permeability assay and resistance described above.

### Pharmacokinetics of DMTS on mice

**Fig. 4:**
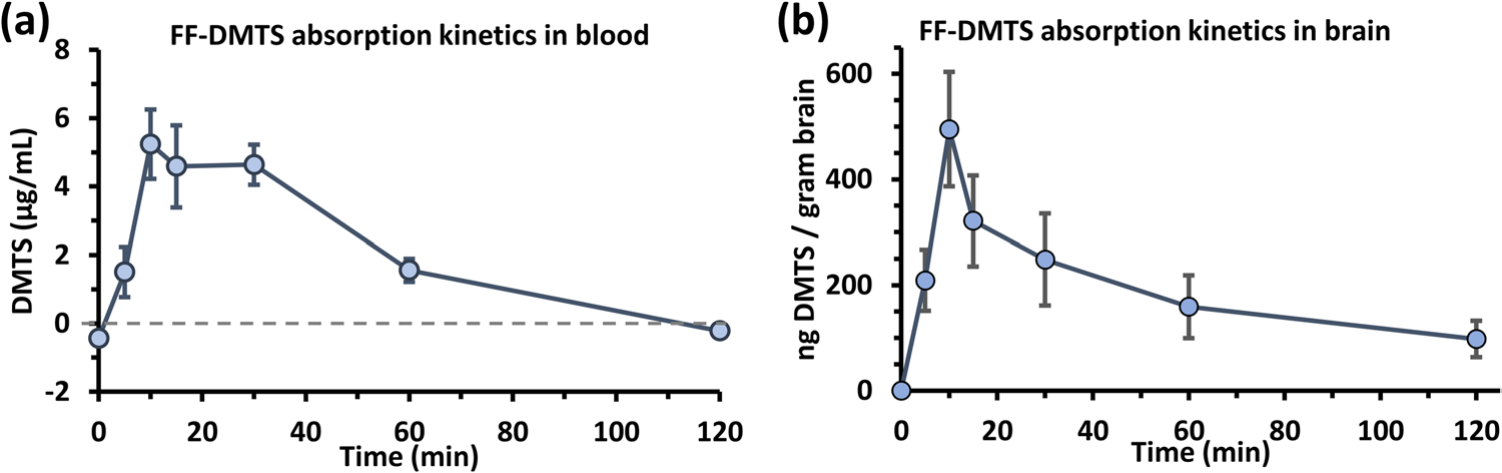
Concentration–time profile of DMTS. The mice were intramuscularly injected with FF-DMTS at 150 mg/kg DMTS dose, then the DMTS concentration was measured in **a** blood and **b** brain. Values are presented as means ± SEM (*n* = 3–4)

In vivo experiments were performed on mice with FF-DMTS to test the pharmacokinetics of DMTS (Fig. 4). Administering 150 mg/kg DMTS dose from FF-DMTS formulation through intramuscular injection resulted in rapid absorption of DMTS in the blood (Fig. 4a). The peak DMTS concentration in plasma was 5.2 ± 1.01 µg/ml which was reached 10 min after injection. The 4.5–5 µg/ml DMTS concentration in plasma was sustained until 30 min, then it decreased to 1.6 ± 0.34 µg/ml after 1 h. In the brain, DMTS peak was reached also after 10 min, which was 495 ± 108 ng/g (Fig. 4b). Then, DMTS concentration quickly dropped to 321 ± 86 and 248 ± 87 ng/g after 15 and 30 min, respectively. A small amount of DMTS (98 ± 35 ng/g) could be observed in the brain even 120 min after the administration.

## Discussion

DMTS is a promising organosulfur molecule to treat CN intoxication [3, 32, 33]. As a lipophilic molecule, a special formulation of DMTS can significantly improve some of its pharmacological properties. Here, we investigated a new formulation of DMTS, called FF-DMTS, and showed its effect on BBB and cellular viability, and we revealed the pharmacokinetic profile of the molecule within this formulation.

The average particle size in the investigated formulation of DMTS was 16 nm. Other solid particles below or around 30 nm have comparable permeability to FF-DMTS through BBB [34], therefore in FF-DMTS, the particles have the ability to cross the BBB. The permeability measurements of FF-DMTS on BBB-PAMPA showed promising results (7.68 × 10^–6^ cm/s). This is similar to drugs which have good in vivo BBB permeability, like caffeine (P_app_ is 11.11 × 10^–6^ cm/s), verapamil (P_app_ is 5.69 × 10^–6^ cm/s), or antipyrine (P_app_ is 10.75 × 10^–6^ cm/s) [35]. Our earlier work with DMTS in polysorbate 80 formulation also showed a good permeability value (11.8 × 10^–6^ cm/s) in BBB-PAMPA model system [18]. The t_LAG_ time with FF-DMTS was 2 min in BBB-PAMPA system, while in polysorbate 80 formulation [18], it was 6.41 min, which means that DMTS in FF-DMTS formulation could pass 3 times faster through the PAMPA barrier than in polysorbate 80 formulation.

The permeability of DMTS through BBB triple co-culture model in this work was 23.8 × 10^–6^ cm/s, which is very high and comparable to the above-mentioned drugs which have good BBB permeability. In the same triple co-culture model, caffeine P_app_ was 64.9 × 10^–6^ cm/s, verapamil P_app_ was 23.4 × 10^–6^ cm/s, and antipyrine P_app_ was 51.8 × 10^–6^ cm/s [35]. The permeability of DMTS in polysorbate 80 formulation through the same culture model of BBB was 158 × 10^–6^ cm/s [18].

The effect of FF-DMTS on the viability of living cells and on the barrier integrity was also investigated. The DMTS had no toxic effect on endothelial cells until 300 µg/ml within 10 min, while longer incubation reduced the impedance of the cell layer. The 10–30 µg/ml FF-DMTS could be applied safely for 10–20 min. Earlier results also showed that 300 µg/ml DMTS in polysorbate 80 formulation does not cause any harmful effect on endothelial cells within 10 min [18]. Furthermore, testing DMTS in another polysorbate 80 formulation did not cause any damage in primary pancreatic acinar cells [14]. However, the treatment with FF-DMTS affected the barrier integrity of the BBB culture model. The cell–cell connections were changed, therefore the TEER values were reduced, the sodium fluorescein permeability increased, and the tight junctions associated protein ZO-1 disappeared in some locations compared to the control untreated group. In our earlier publication [18], the DMTS with polysorbate 80 did not affect either the junctional morphology or the sodium fluorescein permeability. Based on this, it is more likely that the formulation has some effect on the BBB, but further studies with the vehicle should be performed in the future to reveal this phenomenon. Furthermore, during in vivo administration of the FF-DMTS, the vehicle becomes more diluted, resulting in a lower concentration that what was in our in vitro studies. Therefore, any observed effect of the vehicle on the barrier will likely be less pronounced than expected based on this work. It is important to note that the observed reduction in barrier integrity at high concentrations was partial rather than complete on the culture model. Some drugs like antimuscarinic drugs or excipients, such as Cremophore EL or RH40, in the market also reduce the BBB function in vitro, and independently of this those products are safely used in patients [22, 36]. Therefore, considering both the possible remarkable benefits and that side effects related to partial reduction of BBB integrity can be excluded at the peak plasma concentration observed in vivo, as well as the life-threatening nature of CN intoxication, FF-DMTS can be essential and potentially life-saving despite the low possibility of some side effects.

The pharmacokinetics of FF-DMTS in mice were also investigated after 150 mg/kg DMTS administration. The DMTS reached its maximal concentration within 10 min in the serum (5.2 µg/ml) and also in the brain (495 ng/g brain tissue). In our earlier study [18], the mice received a higher dose of DMTS (200 mg/kg) in polysorbate 80, and the DMTS concentration in the serum and brain was, respectively, 26 µg/ml and 1100 ng/g. Although the peak concentration of DMTS is higher following administration in polysorbate 80 formulation (even considering the difference in administration doses), the application of FF-DMTS resulted in sustained DMTS concentrations in the brain. From 10 to 60 min post administration, the concentration of DMTS in the case of FF-DMTS formulation decreased by 32% (from 495 to 335 ng/mg), while when polysorbate 80 formulation was used, the corresponding reduction was 15% (from 1100 to 930 ng/mg) in the brain.

## Conclusions

Overall, we demonstrated that DMTS in FF-DMTS formulation has good blood absorption and excellent brain permeability. The formulation affects the barrier integrity of BBB, without exerting any cell toxicity. The new formulation allows to reach higher DMTS concentration in the product than the previous ones and results in sustained drug concentration in the brain. Based on these findings, we could conclude that FF-DMTS is a good formulation with several advantages, and its further investigation is highly recommended.
